# Lipopolysaccharide induced MAP kinase activation in RAW 264.7 cells attenuated by cerium oxide nanoparticles

**DOI:** 10.1016/j.dib.2015.04.022

**Published:** 2015-05-08

**Authors:** Vellaisamy Selvaraj, Niraj Nepal, Steven Rogers, Nandini D.P.K. Manne, Ravikumar Arvapalli, Kevin M. Rice, Shinichi Asano, Erin Fankenhanel, J.Y. Ma, Tolou Shokuhfar, Mani Maheshwari, Eric R. Blough

**Affiliations:** a Center for Diagnostic Nanosystems, Marshall University, Huntington, WV, USA; b Department of Pharmacology, Physiology and Toxicology, Joan C. Edwards School of Medicine, Marshall University, Huntington, WV, USA; c Department of Cardiology, Joan C. Edwards School of Medicine, Marshall University, Huntington, WV, USA; d Department of Pharmaceutical Sciences and Research, School of Pharmacy, Marshall University, Huntington, WV, USA; e Health Effects Laboratory Division, NIOSH, Morgantown, WV, USA; f Department of Mechanical Engineering and Engineering Mechanics, Michigan Technological University, Houghton, MI, USA

**Keywords:** Sepsis, LPS, MAPK, Raw 264.7 cells

## Abstract

High mortality rates are associated with the life threatening disease of sepsis. Improvements in septic patient survivability have failed to materialize with currently available treatments. This article represents data regarding a study published in biomaterials (Vellaisamy et al., Biomaterials, 2015, in press). with the purpose of evaluating whether severe sepsis mortality and associated hepatic dysfunction induced by lipopolysaccharide (LPS) can be prevented by cerium oxide nanoparticles (CeO2NPs) treatment in male Sprague Dawley rats. Here we provide the information about the method and processing of raw data related to our study publish in Biomaterials and Data in Brief (Vellaisamy et al., Biomaterials, 2015, in press; Vellaisamy et al., Data in Brief, 2015, in press.). The data contained in this article evaluates the contribution of MAPK signaling in LPS induced sepsis. Macrophage cells (RAW 264.7) were treated with a range of cerium oxide nanoparticle concentration in the presence and absence of LPS. Immunoblotting was performed on the cell lysates to evaluate the effect of cerium oxide nanoparticle treatment on LPS induced changes in Mitogen Activated Protein Kinases (MAPK) p-38, ERK 1/2, and SAPK/JNK phosphorylation.

Specifications Table [please fill in right-hand column of the table below]

**Table:** 

Subject area	Biology
More specific subject area	Nanomedicine
Type of data	figure
How data was acquired	Cell culture and immunoblotting
Data format	Raw and analyzed
Experimental factors	Raw 264.7 cells culture in the presence of LPS and CeO2 nanoparticles
Experimental features	Balanced design that encompasses in vitro observations
Data source location	Huntington, WV USA
Data accessibility	*The data presented in this article and is related to*[1,2]

Value of the data

• The data can be referenced by other scientists investigating the effects of LPS on RAW 264.7 cells MAPK signaling.
• The data can provide comprehensive analysis of the effect of cerium oxide nanoparticles on LPS-induced MAPK signaling in cell culture
• These data provides a more thorough understanding of the MAPK involvement in LPS-induced macrophage mediated signaling

## 1 Data, experimental design, materials and methods

### 1.1 Experimental design and methods

This articles contains data related to the research articles entitled “Inhibition of MAP kinase/NF-kB mediated signaling and attenuation of lipopolysaccharide induced severe sepsis by cerium oxide nanoparticles in Biomaterials and Data in Brief article entitled “Cerium oxide nanoparticles inhibit lipopolysaccharide induced MAP kinase/NF-kB mediated severe sepsis” [1,2]. The purpose of this study was to evaluate the contribution of Mitogen Activated Protein Kinase (MAPK) signaling in LPS induced sepsis. For this study, macrophage cells (RAW 264.7) were grown and treated with different concentration of cerium oxide nanoparticle in the presence and absence of LPS. Compared to that seen in the control cells, LPS increased the phosphorylation of p-38, Phosphorylation of ERK 42/44 and Phosphorylation of SAPK/JNK. CeO2 nanoparticle treatment significantly decreased LPS-induced increases phosphorylation of p-38, Phosphorylation of ERK 42/44 and Phosphorylation of SAPK/JNK.

#### 1.1.1 Macrophage culture and uptake of CeO2 NPs and protective effective of CeO2 NPs against LPS

To elucidate the contribution of MAPK signaling in LPS induced macrophage cells, RAW 264.7 cells (ATCC) (1.2×105/ml) were grown in 250 mm culture plate at 30 °C with 5% CO2 in DMEM high glucose medium supplemented with 5% of fetal bovine, containing 1% Pen/Strep (10,000 U Penicillin and 10 mg Streptomycin/ml) and serum until 70–80% confluence and the media was replaced with the fresh medium containing different doses of CeO2 NPs (0, 1, 5, 10, 25, 50, 100 and 1000 ng/ml) for 24 h in the absence and presence of LPS.

#### 1.1.2 Immunoblot analysis of LPS and LPS+CeO2 nanoparticles treated cells

Cells were washed with cold PBS, collected by scraping and centrifuged at 400×*g* for 10 min. Total cell lysates was prepared by cell lytic^TM^ M cell lysis reagent (Sigma) as outlined by the manufacturer. Protein content was estimated in triplicate using the Bradford reagent with bovine serum albumin as a standard. Western blot was performed as mentioned elsewhere. Fifty µg of total protein per well was then subjected to electrophoresis and transfer to nitrocellulose Hybond-C membranes (AmershamTM HybondTM) using standard conditions. Membranes were incubated overnight at 4 °C with the appropriate primary antibody for Phosho p-38, total p-38, Phospho ERK42/44, Phospho SAPK JNK, Total SAPK JNK ( Cell Signaling, Dnavers, MA), washed extensively and then incubated for 1 h at room temperature with a HRP labeled anti-rabbit before detection by ECL (Western Blotting Detection Reagent, GE Health Care Amersham, Piscataway, NJ). Immunoreactive signals were quantified by densitometry using Alpha Innotech software (Santa Clara, California). Beta actin immunoreactivity was used for normalization between samples.

## 2 Results

**Fig. 1:**
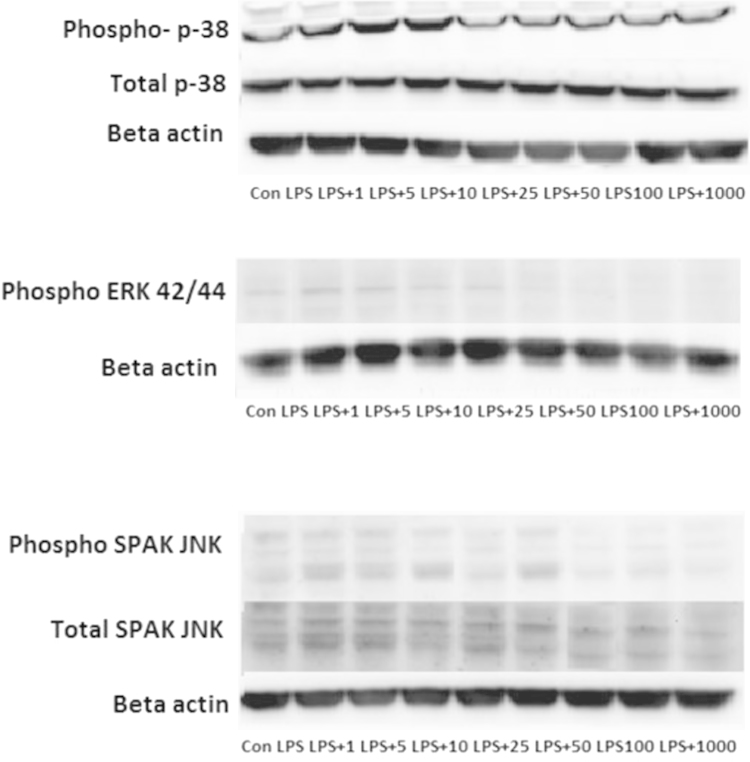
Effect of CeO2 nanoparticles on MAPK activation induced by LPS in RAW cells. Cells were exposed to LPS in the presence and absence of CeO2 nanoparticles for 24 h. Expression of Phospho-p-38 ant total 38, Phospho ERK 42/44 and Phospho SAPK JNK by western blot analysis.

Compared to that seen in the control cells, LPS increased the phosphorylation of p-38, Phosphorylation of ERK 42/44 and Phosphorylation of SAPK/JNK. CeO2 nanoparticle treatment significantly decreased LPS-induced increases phosphorylation of p-38, Phosphorylation of ERK 42/44 and Phosphorylation of SAPK/JNK (Fig. 1).

## Conflict of interests

The authors declare that they have no competing interests.
